# The Late Mr. John W. Davies

**Published:** 1863-10

**Authors:** 


### THE LATE MR. JOHN W. DAVIES.

It is with great regret that we record the death of Mr. John W. Davies, of Princes Street, Leicester Square, Medical Publisher. He died after a brief illness, the sudden termination of an insidious malady. He was a victim to "Bright's disease." An honourable and upright man in all his transactions, he zealously did his best to protect the interests of authors, and to extend the circulation of works entrusted to him. He was the publisher of the present series of this Journal, and his name is retained on the cover of this number, and on the title-page of the volume which the number completes.

